# Association Proceedings

**Published:** 1884-07

**Authors:** 


### THE AMERICAN CLIMATOLOGICAL ASSOCIATION.

The first annual meeting of THE AMERICAN CLIMATOLOGICAL ASSOCIATION was held in Washington, D. C., May 3d and 5th, inst.

In the absence of Prof. Loomis, the President, Dr. F. I. Knight, of Boston, First Vice-President, occupied the chair.

The first day was occupied in adopting a suitable Constitution and By-Laws.

The second day was devoted to the reading of Papers, as follows: Address by the presiding officer, Dr. Knight. "The Aetiology of Pulmonary Phthisis," by Dr .E. F. Eastbrook, of Brooklyn; "The Effects of Sea Air upon Diseases of the Respiratory Organs," by Dr. Boardman Rood, of Atlantic City; "The Relation of Laryngeal Diseases to Pulmonary Diseases," by Dr. F. H. Bosworth, of New York; "Dryness," by Dr. Chas. Dennison, of Denver, Col., in which he dwelt on the following points:—"Variability *versus* Equability.—A rule for classifying climates as to dryness and desirability, based upon low, absolute, and relative humidity and preponderance of sunshine.—The influence of elevation, sunshine, cold, etc., in producing desirable dryness.—The physical effect of dryness on man;" "City Life and City Air Injurious to Consumptives," by Dr. Donaldson, of Baltimore; "The Use of Compressed and Rarefied Air as a Substitute for Change of Climate in the Treatment of Pulmonary Diseases," by Dr. J. Solis Cohen, of Philadelphia.

The following officers were elected for the ensuing year:

*President*—PROF. A. L. LOOMIS, New York.

*First Vice-President*—DR. F. I. KNIGHT, Boston.

*Second Vice-President*—DR. W. H. GEDDINGS, Aiken, S. C.

*Secretary and Treasurer*—DR. J. B. WALKER, Philadelphia.

*Council*—DR. J. H. TYNDALE, New York; DR. E. T. BRUEN, Philadelphia; DR. E. D. HUDSON, New York; DR. FRANK DONALDSON, Baltimore; DR. BEVERLY ROBINSON, New York.

